# Adenosquamous Carcinoma of the Pancreas in a Patient with BRCA2 Mutation: A Case Report

**DOI:** 10.1089/crpc.2015.29003.vye

**Published:** 2015-11-01

**Authors:** Vincent Yeung, Joshua D. Palmer, Noelle Williams, Jonathan C. Weinstein, Danielle Fortuna, Ashwin Sama, Jordan Winter, Voichita Bar-Ad

**Affiliations:** ^1^ Department of Radiation Oncology, Sidney Kimmel Cancer Center, Sidney Kimmel Medical College, Thomas Jefferson University, Philadelphia, Pennsylvania.; ^2^ Department of Radiology, Thomas Jefferson University Hospital, Philadelphia, Pennsylvania.; ^3^ Department of Pathology Anatomy and Cell Biology, Thomas Jefferson University Hospital, Philadelphia, Pennsylvania.; ^4^ Department of Medical Oncology, Thomas Jefferson University Hospital, Philadelphia, Pennsylvania.; ^5^ Department of Surgery, The Jefferson Pancreas, Biliary, and Related Cancer Center, Thomas Jefferson University, Philadelphia, Pennsylvania.

**Keywords:** adenosquamous, BRCA, pancreas, platinum

## Abstract

**Background:** Pancreatic adenosquamous carcinoma (ASC) is an uncommon subtype of pancreatic neoplasm, representing 1–4% of all pancreatic cancers. Given the rarity of this tumor, there is no well-established standard of care regarding treatment. We present the case of a BRCA2-deficient patient who responded tremendously well to a combination of gemcitabine and cisplatin therapy.

**Case presentation:** A 66-year-old Caucasian man presented with a 2-week duration of progressively worsening clay-colored stools, tea-colored urine, and jaundice. Computed tomography scan of the abdomen revealed a 4-cm mass at the head of the pancreas. Preoperative carbohydrate antigen (CA) 19-9 was 255 U/mL (normal <37 U/mL). The patient underwent an uncomplicated pylorus-preserving pancreaticoduodenectomy with pathology revealing 11/12 positive lymph nodes, positive resection margins, perineural invasion, lymphovascular invasion, and positive disease in two distant perihepatic lymph nodes. The patient received one cycle of combination of gemcitabine and abraxane, was subsequently found to be BRCA2 deficient, and completed five cycles of gemcitabine and cisplatin thereafter. CA 19-9 before chemotherapy was 203 U/mL. Postchemotherapy CA 19-9 was 13 U/mL. As of today, the patient continues to do well 22 months postresection without radiographical or gross evidence of disease.

**Conclusion:** Gemcitabine in combination with a platinum agent shows promise in the treatment of pancreatic ASC, particularly in setting of BRCA2 deficiency.

## Introduction and Background

Adenosquamous carcinoma (ASC) of the pancreas is a rare unusual variant of pancreatic cancer. It is estimated to have an incidence of ∼1–4% of all pancreatic malignancies.^1^ Histologically, these tumors consist of two components: glandular adenocarcinoma cells and at least 30% malignant squamous cell carcinoma.^1–3^ Compared to the more common pancreatic histology adenocarcinoma, ASC unfortunately portends a worse prognosis characterized by more aggressive behavior, including larger tumor size and more extensive lymph node involvement.^3^

Given the rarity of ASC, optimal management remains poorly defined, particularly in the postoperative period. Despite aggressive surgical management, reported median overall survivals have ranged from 7 to 14 months.^1–3^ Data regarding adjuvant therapy remain controversial, with the majority of data consisting of anecdotal evidence. Two recent studies have reported conflicting survival data regarding the utility of adjuvant chemotherapy and radiation.^2,3^

In addition, the initial choice of chemotherapy for pancreatic ASC is extrapolated from data regarding pancreatic adenocarcinoma as well as experience from squamous cell cancers, involving other tumor sites such as the esophagus and head and neck.^2^ Adjuvant chemotherapy for pancreatic adenocarcinoma typically consists of gemcitabine either alone or in combination with radiation. Recent data have suggested that the addition of a platinum agent to adjuvant regimens for resected pancreatic ASC may improve survival among high-risk patients.^2^

We present the case of a patient with pancreatic ASC who was found to be BRCA2 deficient and had a remarkable response to combination of gemcitabine and cisplatin chemotherapy in the adjuvant setting.

## Presentation of Case

**FIG. 1.**
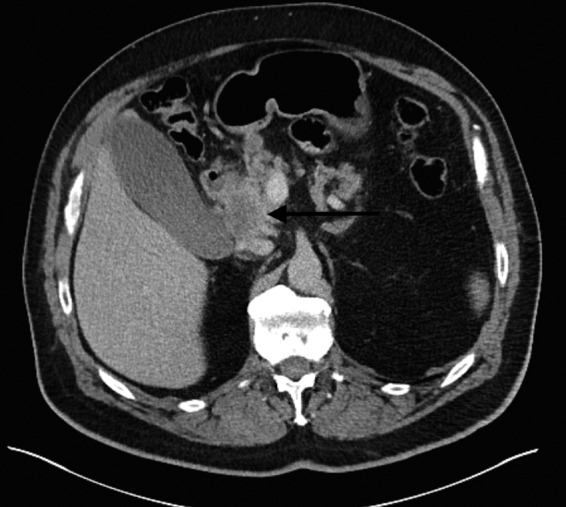
Axial contrast-enhanced computed tomography (CT) image demonstrates a defined hypo-attenuating mass in the head of the pancreas (*arrow*), which abuts the second portion of the duodenum.

**FIG. 2.**
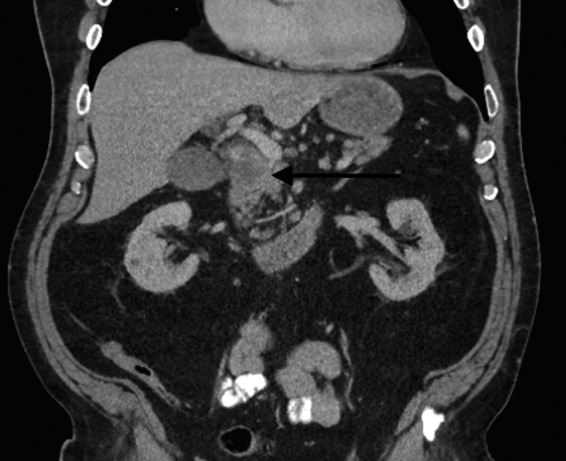
Coronal contrast-enhanced CT image demonstrates the hypo-attenuating mass in the head of the pancreas (*arrow*).

The patient is a 66–year-old man with a medical history significant for hyperlipidemia and hypertension, who presented with 2 weeks of progressively worsening clay-colored stools, tea-colored urine, and jaundice. There was a family history of pancreatic cancer in the patient's maternal grandmother, as well as a sister with cancer of unknown origin, uterine cancer in the mother, and bladder cancer in the patient's father. On presentation, physical examination was notable for scleral icterus and jaundice. There were no palpable abdominal masses and no hepatosplenomegaly. A computed tomography (CT) scan of the abdomen revealed an ill-defined, low attenuation mass at the head of the pancreas, measuring 3.0 × 2.7 cm (Figs. 1 and 2). There was no radiographical or gross evidence of metastatic disease on the preoperative staging work up. His peripheral laboratories revealed a total bilirubin of 10 mg/dL (0.3–1.9 mg/dL), whereas the aspartate aminotransferase and alanine aminotransferase were 249 IU/L (8–48 IU/L) and 618 IU/L (7–55 IU/L), respectively. Alkaline phosphatase was elevated at 242 IU/L (45–115 IU/L). The patient's hemoglobin was normal at 13 g/dL. Carbohydrate antigen (CA) 19-9 on presentation was 255 U/mL (<37 U/mL).

**FIG. 3.**
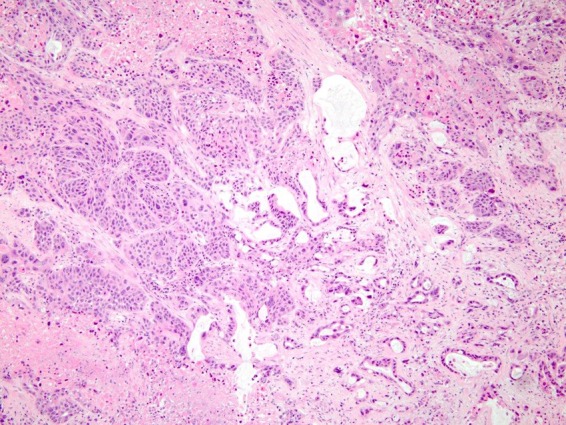
Adenosquamous carcinoma (hematoxylin and eosin stain; 100× magnification). The tumor has both malignant glandular (adenocarcinoma, *right*) and squamous (squamous carcinoma, *left*) components.

An esophagogastroduodenoscopy and endoscopic ultrasound were subsequently performed, which revealed a 4-cm mass invading the duodenum, consistent with pancreatic cancer. Biopsy revealed poorly differentiated adenocarcinoma of the pancreas. A pylorus-preserving pancreaticoduodenectomy was performed. At the time of surgery, the tumor was found to be 3.5 cm in the greatest diameter. Final pathology revealed ASC of the pancreas (Fig. 3). The patient was found to have 11/12 specimen lymph nodes positive for metastatic cancer, along with two positive perihepatic lymph nodes that were distant from the site of the primary tumor. In addition, surgical resection margins were positive at the uncinate process and pancreatic neck. Lymphovascular invasion and perineural invasion were present. The patient did well postoperatively and was discharged home on postoperative day 8. Postoperative CA 19-9 was 203 U/mL.

As part of an institutional study, the patient was referred for genetic testing. The patient received one cycle of combination of gemcitabine and abraxane before he was found to have a BRCA2 mutation. Abraxane was changed to cisplatin and he completed five cycles of gemcitabine and cisplatin without major complications. Eighteen months after initial diagnosis, a repeat CT scan of the chest, abdomen, and pelvis revealed no evidence of disease as well as a normal CA 19-9 of 13 U/mL. The patient continues to do well at the time of publication.

## Discussion

Pancreatic ASC is a rare variant of pancreatic cancer that is associated with a particularly poor prognosis. Wild et al. have recently published data on 62 patients with ASC of the pancreas and showed that the prognostic factors associated with inferior outcome included lack of adjuvant therapy, positive resection margins, lymph node involvement, and age greater than 65 years. A second multivariate analysis, including only the patients with pancreatic ASC who received adjuvant therapy, revealed that the omission of a platinum agent in the adjuvant regimen and a larger tumor diameter were independent predictors of inferior survival.^2^

Our patient is unique in that despite multiple poor prognostic factors, including his age, positive regional and distant lymph nodes, positive resection margins, and large tumor diameter, he responded remarkably well to gemcitabine and cisplatin chemotherapy. When the two agents are delivered together, there appears to be a synergistic effect.

In addition, our patient was found to have a mutation in a BRCA2 gene. Studies on BRCA2-mutated ovarian cancer have shown improved survival and improved chemotherapy response with platinum agents in other disease sites such as ovarian cancer and pancreatic cancer.^4^ It is believed that as a result of the BRCA2 mutation, DNA double-strand repair through homologous recombination is impaired. Consequently, the DNA cross-links introduced by agents such as cisplatin lead to genome instablility.^4^ Our patient's tumor was BRCA2 deficient and was apparently especially sensitive to the combination of gemcitabine and cisplatin chemotherapy in the adjuvant setting.

## Conclusion

Gemcitabine in combination with a platinum agent shows promise in the treatment of pancreatic ASC and should be considered in adjuvant chemotherapy selection for this group of patients. Furthermore, the significance of BRCA2 mutation in pancreatic cancer prognosis and response to platinum-containing chemotherapy regimens and poly (ADP-ribose) polymerase inhibitors are being investigated in clinical trials.

## Abbreviations Used

ASC: adenosquamous carcinoma
CA: carbohydrate antigen
CT: computed tomography
